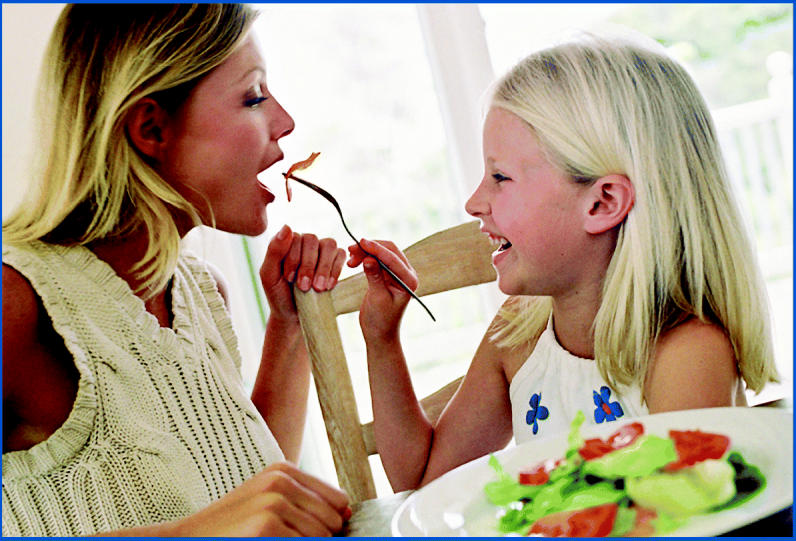# Headliners: Maternal Nutrition and Child Cancer: Mother’s Pre-pregnancy Diet May Influence Child Cancer Risk

**Published:** 2004-11

**Authors:** Susan M. Booker

Jensen CD, Block G, Buffler P, Ma X, Selvin S, Month S. 2004. Maternal dietary risk factors in childhood acute lymphoblastic leukemia (United States). Cancer Causes Control 15(6):559–570.

Acute lymphoblastic leukemia (ALL) is the most common childhood cancer (with 2,400 cases diagnosed each year in those under age 20) and the second most common cause of mortality in children aged 1–14. Recent research has confirmed that ALL can originate *in utero*. New findings from the NIEHS-funded Northern California Childhood Leukemia Study (NCCLS) show that the disease may originate even earlier—in the foods a woman eats before she even becomes pregnant.

The effect of maternal diet on child leukemia risk has not been rigorously studied; the few studies that have been done have focused on specific dietary factors, and the results have been mixed. The NCCLS is a population-based case–control study of risk factors for child leukemia, including maternal diet. It is the first study to capture mothers’ overall dietary patterns and relate them to child leukemia.

Researchers compared 138 mothers of children diagnosed with ALL with a control group of 138 mothers whose children did not have cancer. All the mothers completed a questionnaire pertaining to their diet in the 12 months prior to pregnancy. The researchers chose this period as a more accurate reflection of each woman’s typical diet, compared to pregnancy, when diet can vary with the degree of nausea experienced. The questionnaire asked about 76 food items, plus use of vitamins, certain reduced-fat foods, and cooking fat.

The researchers found that the more vegetables, fruits, and proteins a woman ate, the lower the risk of her child having leukemia. Of the vegetables and fruits, carrots and cantaloupe showed the highest inverse effect, perhaps because of these foods’ high carotenoid content. String beans and peas also correlated inversely with ALL risk. Among the proteins, beef and beans—both sources of the antioxidant glutathione—showed the highest inverse effect. Use of vitamin supplements was not significantly linked to leukemia risk.

The authors stress that dietary factors work together, and no one food should be singled out in attributing risk or benefit. Further, a cause-and-effect relationship can not be concluded from this study. However, they write, it remains prudent for women who are pregnant or think they may become pregnant to eat a diet rich in fruits and vegetables.

## Figures and Tables

**Figure:**